# Effects of Match Location, Quality of Opposition and Match Outcome on Match Running Performance in a Portuguese Professional Football Team

**DOI:** 10.3390/e23080973

**Published:** 2021-07-29

**Authors:** José E. Teixeira, Miguel Leal, Ricardo Ferraz, Joana Ribeiro, José M. Cachada, Tiago M. Barbosa, António M. Monteiro, Pedro Forte

**Affiliations:** 1 Research Centre in Sports Sciences, Health and Human Development, 5001-801 Vila Real, Portugal; rmpf@ubi.pt (R.F.); barbosa@ipb.pt (T.M.B.); mmonteiro@ipb.pt (A.M.M.); pedromiguel.forte@iscedouro.pt (P.F.); 2 Department of Sports, Exercise and Health Sciences, University of Trás-os-Montes e Alto Douro, 5001-801 Vila Real, Portugal; 3 Departamento de Desporto e Educação Física, Instituto Politécnico de Bragança, 5300-253 Bragança, Portugal; 4 Department of Sports, Douro Higher Institute of Educational Sciences, 4560-708 Penafiel, Portugal; amnfla@gmail.com (M.L.); joana.ribeiro@iscedouro.pt (J.R.); mario.cachada@iscedouro.pt (J.M.C.); 5 Department of Sports Sciences, University of Beira Interior, 6201-001 Covilhã, Portugal

**Keywords:** physical performance, activity profile, time-motion, match analysis, team sports

## Abstract

The aim of this study was to analyze the effects of match location, quality of opposition and match outcome on match running performance according to playing position in a Portuguese professional football team. Twenty-three male professional football players were monitored from eighteen Portuguese Football League matches during the 2019–2020 season. Global positioning system technology (GPS) was used to collect time-motion data. The match running performance was obtained from five playing positions: central defenders (CD), fullbacks (FB), central midfielders (CM), wide midfielders (WM) and forwards (FW). Match running performance was analyzed within specific position and contextual factors using one-way analysis of variance (ANOVA) for repeated measures, standardized (Cohen) differences and smallest worthwhile change. CM and WM players covered significantly greater total distance (F = 15.45, *p* = 0.000, η^2^ = 0.334) and average speed (F = 12.79, *p* < 0.001, η^2^ = 0.294). WM and FB players covered higher distances at high-speed running (F = 16.93, *p* = 0.000, η^2^ = 0.355) and sprinting (F = 13.49; *p* < 0.001, η^2^ = 0.305). WM players covered the highest number of accelerations (F = 4.69, *p* < 0.001, η^2^ = 0.132) and decelerations (F = 12.21, *p* < 0.001, η^2^ = 0.284). The match running performance was influenced by match location (*d =* 0.06–2.04; CI: −0.42–2.31; SWC = 0.01–1.10), quality of opposition (*d =* 0.13–2.14; CI: –0.02–2.60; SWC = 0.01–1.55) and match outcome (*d =* 0.01–2.49; CI: −0.01–2.31; SWC = 0.01–0.35). Contextual factors influenced the match running performance with differential effects between playing positions. This study provides the first report about the contextual influence on match running performance in a Portuguese professional football team. Future research should also integrate tactical and technical key indicators when analyzing the match-related contextual influence on match running performance.

## 1. Introduction

Football is an intermittent team sport characterized by high physiological demands [1]. Elite players were found to cover 9–14 km in total during an official football match [2,3]. The high-intensity activity (>19.8 km·h^–1^) represents 8–10% of the total distance completed, since most movement activities are carried out in low-intensity zones [4,5]. The distances covered at high intensities are a valid indicator to evaluate physical performance in professional football given its relationship with the training process [6,7]. High-speed running, sprints, tackles, impact accelerations and decelerations occur intermittently in a match-play, which require greater physiological and neuromuscular demands [8].

Researchers’ and practitioners’ interest in the physical performance has been growing over the last four decades at the professional football level [9,10]. Monitoring players’ work-rate profiles during training and competition has become more practicable with computer-aided time-motion approaches [5,6,7]. Additionally, using tracking systems to monitor match demands has become a hot topic of research, referring to work rate, activity profile or match running performance [5,9,11,12]. Several studies quantified the match running performance across national professional leagues, such as the English [13,14,15,16,17,18,19,20,21], Italian [3,22,23], Spanish [19,24,25,26], French [20,27,28], German [29,30,31,32], Brazilian [33,34], Norwegian [35,36], Danish [37] and Australian leagues [38,39]. The literature also focused on the European Champions League [40,41,42,43,44], UEFA Cup/Europe League [41,44] and the World Cup [45,46,47]. Current research has also demonstrated an influence of position on the players’ match demands [15,19,25,26,48], and further, the football game’s evolution has demonstrated a position-specific physical increase over time [11,49,50]. Generally, central midfielders covered more distance, and wide midfielders covered more distance at high-intensity zones [13,51]. The central defenders and wide defenders covered more distance at low-intensity zones [51]. Forwards sprint significantly less frequently than central defenders [21]. Central defenders performed significantly fewer explosive and leading sprints [13]. Accelerations contributed to 7–10% of the player workload for all playing positions during a match-play, while decelerations represented 5–7% [52].

Nevertheless, interpreting match running performance should consider the influence of contextual, environmental or situational factors [24,53,54,55]. Studies have pointed to a strong influence of contextual factors on the match running performances from top football national leagues and continental competitions [24,30,56,57,58,59,60,61,62,63,64]. Hence, independent and interactive potential effects have been reported for match running performance according to match location, quality of opposition and match status in professional football [59,65]. Contextual factors have a potential influence on the relationship between match running and the overall performance dimension [55]. Thus, match running performance shall be adjusted according to the intended contextual factors underlying the match-play [24,53,55]. Indeed, elite players normally cover less high-intensity distances when winning [66]. Total distance covered by players was found to be higher when playing at home and against high-ranked teams [24,59]. Linking players’ behaviors and match outcomes in specific contexts has been identified as a crucial insight to develop specific game strategies or training designs [11].

To the best of our knowledge, no studies have analyzed the influence of contextual factors on match running performance in a professional Portuguese football competition. Therefore, the aim of this study was to analyze the effects of match location, quality of opposition and match outcome on match running performance according to playing position in a Portuguese professional football team. It was hypothesized that the contextual factors and specific playing positions influence the match running performance.

## 2. Materials and Methods

### 2.1. Participants and Match Sample

Twenty-three male professional football players (age: 32.02 ± 1.19 years; height: 1.82 ± 0.01 m; weight: 74.74 ± 0.53 kg) participated in eighteen Portuguese Second League (Leadman LigaPro^®^,Lisbon, Portugal) matches (8 home and 10 away) during the 2019–2020 season. The sampled players were characterized to one of five playing positions (goalkeeper was excluded): central defenders (CD), fullbacks (FB), central midfielders (CM), wide midfielders (WM) and forwards (FW). The numbers of subjects in the different subgroups were: CD (*n* = 6), FB (*n* = 4), CM (*n* = 5), WM (*n* = 5) and FW (*n* = 3). The playing positions were organized into ten dyads: CD vs. FB, CD vs. WM, CD vs. CM, CD vs. FW, FB vs. WM, FB vs. CM, FB vs. FW, CM vs. WM, CM vs. FW and WM vs. FW. The match data correspond to the observations of the seven outfield players for each match in the same team (*n* = 128). The analysis has only considered the players who were part of the starting line-up and performed the entire match duration. The substituted players and non-starting players were not analyzed. The number of observations per position role was: CD (*n* = 36), FB (*n* = 31), CM (*n* = 33), WM (*n* = 19) and FW (*n* = 9). The match data showed 3 wins, 9 draws and 5 loses, with a total of 13 goals scored and 15 goals conceded by the sampled team. The matches (2 × 45′) were performed in official stadiums (FIFA standard, natural grass, ~100 × 70 m), between 10:00 AM and 08:00 PM, and the mean environment temperature was 14.9 ± 5.3 °C.

All participants were informed about the aim and risks in the investigation. The study includes only the players that have signed the informed consent, and was conducted according the ethical standards of the Declaration of Helsinki. The experimental approach was approved and followed by the Technical and Scientific Board of the Douro Higher Institute of Educational Sciences.

### 2.2. Data Collection and Procedures

The seven main players were monitored in each match using a portable GPS throughout the whole match duration (STATSports Apex^®^, Newry, Northern Ireland). The GPS device provides raw position velocity and distance at 10 Hz sampling frequencies, including accelerometer (100 Hz), magnetometer (10 Hz) and gyroscope (100 Hz). Each player wore the micro-technology inside a mini-pock of a custom-made vest supplied by the manufacturer, which was placed on the upper back between both scapulae. All devices were activated 30 min before the match data collection to allow an acceptable clear reception of the satellite signal. Respecting the optimal signal to the measurement of human movement, the match data considered eight available satellite signals as the minimum for the observations [67]. The validity and reliability of the global navigation satellite systems (GNSS), such as the GPS tracking, have been well-established in the literature [67,68,69]. The current variables and thresholds have a small error of around 1–2% reported for the 10 Hz STATSports Apex^®^ devices [68].

### 2.3. Contextual Factors

Contextual factors were codified based on three independent variables: match location, quality of opposition and match outcome. These contextual dimensions have been extensively documented in the literature [54,65]. Match location was split into “home” and “away”, based on when the team under analysis was playing at home or away. Quality of opposition was classified from “high-ranking” (i.e., from 1st to 5th position in the league ranking), “medium-ranking” (i.e., from 6th to 12th position in the league ranking) and “low-ranking” (i.e., from 13th to 18th position in the league ranking). Quality of opposition was classified according to the final standing of the 2019–2020 season. Match outcome was analyzed according to “lose”, “draw” or “win” at the end of the match-play.

### 2.4. Physical Load Measures

The match running performances were obtained with the following time-motion data using physical load measures: total distance (TD) covered (m), average speed (AvS) expressed in distance covered per minute (m·min^−1^), high-speed running (HSR) distance (m), number of sprints (SPR), number of accelerations (ACC) and number of decelerations (DEC). The GPS software only provided information about the locomotor categories above 19.8 km·h^−1^: HSR (19.8–25.1 km·h^−1^) and SPR (>25.1 km·h^−1^). Both acceleration variables (ACC and DEC) considered the movements made in the maximum intensity zone (3 m·s^−2^): ACC (>3 m·s^−2^) and ACC (<3 m·s^−2^). The high-intensity activity thresholds were adapted from previous studies [6,7].

### 2.5. Statistical Analysis

For descriptive statistics, the Kolmogorov–Smirnov and Levene’s tests were used to test the normality and homogeneity, where a normal distribution was observed. Differences between playing positions, contextual factors and match running performance were tested with one-way analysis of variance (ANOVA) for repeated measures. When a significant difference occurred, Bonferroni post-hoc tests were used to identify localized effects. Dunnett’s T3 post-hoc tests were applied if variances were not homogeneous. Bonferroni post hoc was performed to evaluate TD, rHSR, SPR and AvS. The Dunnett’s T3 post-hoc was executed for ACC and DEC.

Standardized effect sizes (ES) were calculated by Cohen’s d, and the thresholds were classified as: 0.2, trivial; 0.6, small; 1.2, large; >2.0, very large [70,71]. Smallest worthwhile change (SWC) was calculated as 0.2 multiplied by standard deviation (SD). Additionally, trivial area was calculated from the SWC determined as 0.2 times the between-playing positions [72].

Statistical significance was set at *p* < 0.05. Data are presented as the mean ± SD. Mean differences (Δ) are presented in absolute values. All statistical analyses were conducted using IBM SPSS Statistics for Windows (Version 27.0., IBM Corp, Armonk, NY, USA). ES calculations were performed with G*Power (Version 3.1.5.1 Institut für Experimentelle Psychologie, Düsseldorf, Germany). Data visualization was produced using GraphPad Prism (GraphPad Software, Inc., San Diego, CA, USA).

## 3. Results

### 3.1. Effects of Contextual Factors on Match Running Performance

The descriptive statistics of match running performance according to competitive stage, match location quality of opposition and match outcome are presented in Table 1.

**Figure 1:**
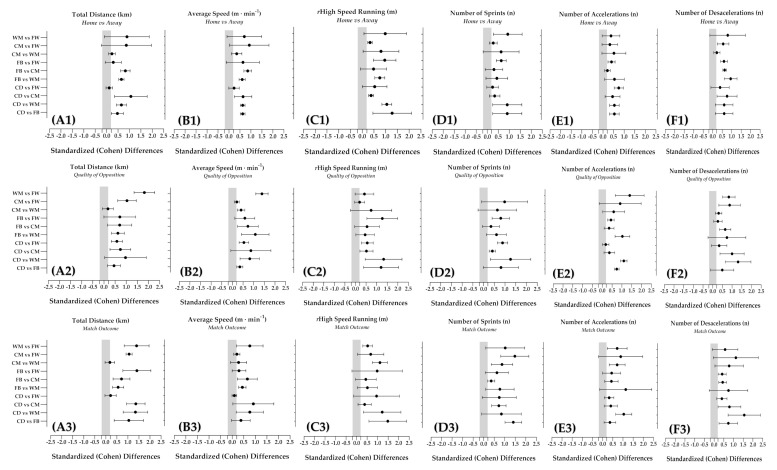
The effects of contextual factors on match running performance according to playing positions were reported using standardized (Cohen) differences, following the match location (**A1**–**F1**)**,** quality of opposition (**A2**–**F2**) and the match outcome (**A3**–**F3**). Trivial area was calculated from the smallest worthwhile change determined as 0.2 times the between-playing positions. Abbreviations: CD—central defenders; CM—central midfielders; DEC—decelerations; FB—fullbacks; FW—forwards; Km—kilometers; m—meters; m · min^−1^—meters per minute; n—number; WM—wide midfielders.

Table 2, Table 3 and Table 4 present the influence of contextual factors on match running performance according to playing positions. Standardized (Cohen) differences, 95% CI and SWC for each contextual factor are presented in Figure 1. Match running performance was influenced with trivial to very large effects by match location (*d =* 0.06–2.04; CI: −0.42–2.31; SWC = 0.01–1.10), quality of opposition (*d =* 0.13–2.14; CI: −0.02–2.60; SWC = 0.01–1.55) and match outcome (*d =* 0.01–2.49; CI: −0.01–2.31; SWC = 0.01–0.35). Quality of opposition’s influence had a very large effect on TD for WM vs. FW (*d* = 2.14, CI: 1.88–2.40; SWC = 0.30). Match outcome had a very large effect on rHSR for CD vs. FB (*d* = 2.12, CI: 1.97–2.27; SWC = 0.17) and CD vs. WM (*d* = 2.49, CI: 2.38–2.60; SWC = 0.13). CD vs. WM also showed a very large result of the quality of the opposition’s influence for DEC (*d* = 2.14, CI: 1.97–2.31; SWC = 0.19). 

### 3.2. Effects of Playing Position on Match Running Performance

The descriptive statistics of match running performance of each playing position were presented in Table 5. The match running performance was influenced by playing position on all physical load measures analyzed: TD (F = 15.45, *p* < 0.001, η^2^ = 0.334), AvS (F = 12.79, *p* < 0.001, η^2^ = 0.294), rHSR (F = 16.93, *p* < 0.001, η^2^ = 0.355), SPR (F = 13.49, *p* < 0.001, η^2^ = 0.305), ACC (F = 4.69, *p* < 0.001, η^2^ = 0.132) and DEC (F = 12.21, *p* < 0.001, η^2^ = 0.284).

**Figure 2:**
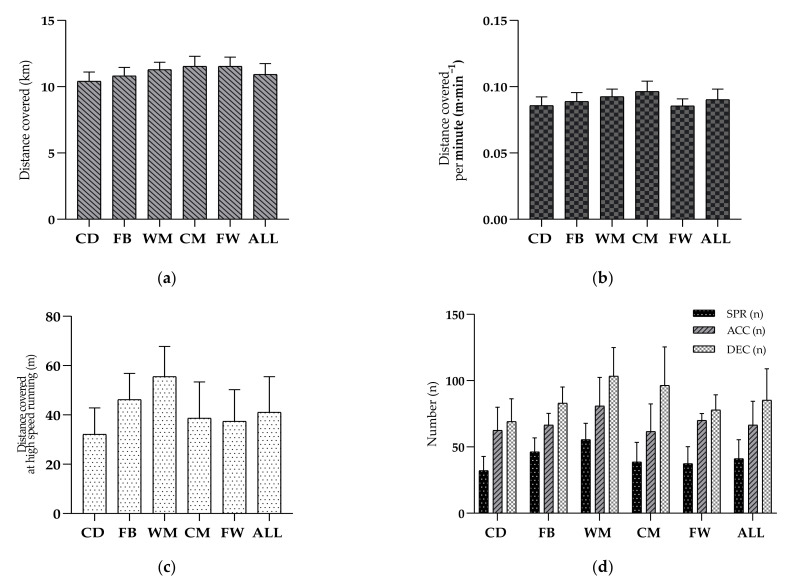
Match running performance according to playing position: (**a**) total distance covered (km), (**b**) average speed expressed in distance covered per minute (m · min^−1^), (**c**) distance at relative high-speed running (m) and (**d**) number of sprints, accelerations and decelerations. Abbreviations: ACC—accelerations; ALL—overall independent position group; AvS—average speed; CD—central defenders; CM—central midfielders; DEC—decelerations; FB—fullbacks; FW—forwards; km—kilometers; m—meters; m · min^−1^—meters per minute; n—number; rHSR—relative high-speed running; SPR—sprints; WM—wide midfielders.

Specifically, the pairwise comparisons for the playing position factor revealed (see Figure 2) that CM and WM players covered significantly greater TD than other playing positions: CM vs. CD (Δ = 1119.09 m, *p* < 0.001, *d* = 1.54), CM vs. FB (Δ = 714.90 m, *p* < 0.001, *d* = 1.03), CM vs. FW (Δ = 1266.87 m, *p* < 0.001, *d* = 1.74), and WM vs. CD (Δ = 887.37 m, *p* < 0.001, *d* = 1.41), WM vs. FB (Δ = 473.17 m, *p* < 0.001, *d* = 0.80) and WM vs. FW (Δ = 551.97 m, *p* < 0.001, *d* = 1.64).

Regarding the distance covered per minute, CM players covered significantly more distance than any other playing position except WM players: CD (Δ = 0.11 m · min^−1^, *p* = 0.000, *d* = 11.80), FB (Δ = 0.01 m·min^−1^, *p* < 0.001, *d* = 12.23) and FW (Δ = 0.01 m · min^−1^, *p* = 0.000, *d* = 12.67). WM players covered significantly more rHSR than all playing positions (Δ = 351.14 m, *p* < 0.001, *d* = 1.38–2.09), except FB players. FB players covered significantly greater rHSR than CD (Δ = 346.88 m, *p* = 0.001, *d* = 1.60), CM (Δ = 184.08 m, *p* = 0.003, *d* = 0.89) and FW (Δ = 242.97 m, *p* = 0.016, *d* = 1.28).

WM players presented significantly higher SPR than all playing position except FB players: CD (Δ = 23.39, *p* < 0.001, *d* = 2.05), CM (Δ = 16.85, *p* = 0.000, *d* = 1.25) and FW (Δ = 18.14, *p* = 0.003, *d* = 1.45). The SPR distance covered by FB players was significantly greater than CD (Δ = 14.06, *p* < 0.05, *d* = 1.33). FW players showed significantly lower SPR values compared to CM (Δ = 1.28, *p* < 0.05, *d* = 0.09).

WM players covered significantly higher ACC than CM players (Δ = 19.23, *p* < 0.001, *d* = 0.91) and CD players (Δ = 18.37, *p* = 0.002, *d* = 0.93). WM players covered significantly greater DEC than other playing positions, except CM players: CD (Δ = 34.25, *p* < 0.001, *d* = 1.88), FB (Δ = 20.36, *p* < 0.05, *d* = 1.14) and FW (Δ = 25.53, *p* < 0.05, *d* = 1.48). FB players presented significantly higher than CD players (Δ = 13.90, *p* < 0.05, *d* = 0.94).

## 4. Discussion

The aim of this study was to analyze the effects of match location, quality of opposition and match outcome on match running performance according to playing position in a Portuguese professional football team. In general, our findings described significant differences between playing positions considering the match running performance. As hypothesized, the findings confirmed the influence of match location, quality of opposition and match outcome on match running performance, with some differences according to playing position.

### 4.1. Contextual Factors and Their Influence on Match Running Performance

The present study confirmed the influence of match location (trivial to large effects), quality of opposition (trivial to very large effects) and match outcome (trivial to very large effects) on match running performance. Additionally, our findings reported a match-related contextual influence with a specific position dependence. Very large effects were found for match outcome and quality of opposition in TD, rHSR and DEC, with positional differences (i.e., WM vs. FW, CD vs. FB and CD vs. WM). Previous studies have also verified these positional differences on the match running performance depending on the contextual factors [11,24,53,54,55]. High-intensity activity differences were highly influenced, with forwards more active when winning and vice versa for defenders [53]. This specific position dependence was also reported by Aquino et al. [73], who reported a higher relative contribution to the variance in high-intensity activities in Brazilian professional football players. Bush et al. [61] described a higher match-to-match variability for central defenders and wide midfielders in the HSR and SPR demands. This positional effect was also reported in different odds of winning according to playing position [70]. In this sense, Tucker et al. [63] mentioned an advantage to home-winning and home-goal percentage. Relating to effects of match location, previous studies have observed that the teams win most when playing at home [59]. TD seems to be the most affected, and high-intensity distances were covered when winning [66]. García-Unanue et al. [60] also reported an impact of match location on physical performance, whereby playing away showed the highest distance covered in the second half. This likely happens because players show higher levels of synchronization as the match develops [74]. In addition, the player’s usually covered less high-intensity activity when winning than when losing or drawing [24]. Indeed, it seems that more organized teams showed a greater relational capacity between their players, who therefore do not need to run as much [75,76]. Another reason could be outlined by fatigue effects, causing different pacing strategies and team coordination [77,78,79]. Opponent level was reported in previous investigations as an important contextual factor [4,29,59]. Higher ranked teams covered more distance at walking and jogging speeds [59]. Additionally, higher ranked teams covered less TD and less HSR compared to lower-ranked teams, among which higher total distance was performed at home and against high-ranked teams [24,59]. Our findings suggested that quality of opposition and match outcome have a greater influence than match location. Additionally, the contextual factors and their changes seem to differ between playing positions. These differences in the effects of contextual factors can be considered to control the weekly training load and adequately taper the strategy in preparation for the next match-play [6,7].

### 4.2. Match Running Performance

Positional differences on match running performance were reported in this study. CM and WM players covered significantly greater TD than other playing positions (strong effect). Previous studies also reported that midfielders covered longer distances in comparison to defenders and forwards [4,14,15,19,20,23,25,26,40,44]. The midfielder positions covered a 3% longer distance than forwards, and 7% longer than that achieved by the defenders [44]. In contrast, other studies only reported differences in the CD and/or FW players [4,19,80,81]. The contrasting findings may be explained by the differences in the match running performance according to competition standards [20]. Hence, it is important to compare the performed match running in a Portuguese second division with other professional football leagues. TD observed in our study (11,539.09 m) differed from other national leagues, such as the English Premier League (10,451–10,746 m) [15,19], Italian ‘Serie A’ (8943.0–10,330 m) [3,22], English Championship League (11,102 m) [17], Spanish ‘La Liga’ (5667–11,393 m) [24,25,26], German ‘Bundesliga’ (11,621 m) [29], French ‘Ligue 1’ (10,746–12,029 m) [20,27], Norwegian League (11,230 m) [35], Danish ‘Superliga’ (10,776 m) [37], Australian ‘A League’ (10,100–10,274 m) [38,39] and Brazilian ‘Serie A’ (10,012 m) and Brazilian lower divisions (8518–9375 m) [80,82]. Indeed, it appears evident that there is a trend to cover longer distances in the lower divisions. It is possible that higher levels of collective synchronization allow a greater individual and inter-individual capacity to explore space and interpret match information [74].

Concerning the distance covered per minute, CM and WM covered significantly greater AvS than other playing positions (very large effect). When compared to the distance covered across different playing positions, the literature reported a similar frequency in the distance covered at lower intensities [38]. Therefore, examining the high-intensity activity provides a valid insight into physical performance with their strong training status [9,10]. Our findings demonstrate that WM players covered significantly more rHSR than all playing positions, except FB players (moderate to large effect). FB players covered significantly greater rHSR than CD, CM and FW players (moderate to large effect). Our findings are consistent with other reports [14]. Previous studies also achieved lower HSR distances covered by CD players [36,83]. Additionally, one study also confirmed the greatest HSR distances for WM players, however the CM players presented higher jogging and running distances (7.2–19.7 km · h^−1^) [6]. In our match data, the SPR values showed significant differences between FB vs. CD (moderate effect) and FW vs. CM (small effect). The FW players sprinted less than the CM players. In contrast, other studies showed greater SPR distances for FW and FB than CM and CB players [3,4,22]. Dellal et al. [20] reported a greater SPR distance for FW compared to CD and FB. Di Salvo et al. [41] also reported that CD players sprinted less, however, the authors achieved the greatest SPR distance for WM players. Here, our findings demonstrate that match performance is crucial to explain the specific demands placed on each playing position. Moreover, there are other important factors to consider in the analysis of high-intensity movements which may have influenced our findings. First of all, there is a documented match-to-match variation in the high-intensity activity [84,85]. The literature reports differences in the performed match running through the two halves or period bouts [18,19,27,35,80]. Understanding positional differences on match running performance can help coaches to better plan and periodize on the basis of these match-to-match variations.

Regarding the performed acceleration profiles, our study reported a higher ACC in the WM players than CD and CM (small effect). Additionally, WM players presented higher DEC than other playing positions (moderate to large effect). Our results were not consistent with the literature, which documents positional differences in the acceleration profiles during competitive matches [14,26,37]. The players in lateral positions accelerated more than central positions [35]. Our findings had substantially less ACC and DEC than previously reported findings in higher-ranked leagues [26,35,37,39]. On the other hand, the average number of maximal accelerations per match and peak acceleration achieved during the match-play does not seem to be influenced by positional roles [14]. However, these studies generally use different acceleration thresholds that could bias the ACC and DEC outputs upon the time-dependent and transient reductions [13]. Ideally, future studies should also consider the ACC and DEC through each half of the match. Normally, the players performed lower numbers of ACC and DEC in the second half than the first half [14,18,35,37].

### 4.3. Limitations and Future Perspectives

Our study has some limitations, which means that the results should be interpreted with caution: (i) match data did not consider the different period bouts and halves of the match, as in other studies [13,14,15,17,18,20,48], and (ii) technical factors (i.e., running with or without the ball) [56,86,87], tactical key indicators (i.e., possession strategies) [65,83,88] and collective behavior must be considered for a more ecological analysis [9,10,74,75,76,77,78,79]. (iii) The different methodological approaches used in the literature should be considered when interpreting our findings [6,7], (iv) cumulative effects of a pre-match training and players’ cognitive status prior to match-play were not controlled in this research [89,90,91] and (v) our match data reflect only one team and hence cannot be extended to all Portuguese professional teams. Hence, more analyses are required for this purpose, with a wider follow-up. Future research should also consider the relationship of accumulated training load, such as congested fixture, players’ starting status and competitive level [6,92]. Match running performance analysis should also include the game model, pacing strategies and collective tactical behavior [9,55,77,78,79].

## 5. Conclusions

This study confirmed that the match running performance was influenced by playing positions and match-related contextual factors. Additionally, this study provides the first report about the contextual influence on match running performance in a Portuguese professional football team. There was an influence of match location, quality of opposition and match outcome. Our match data suggest that positional differences and contextual factors are important factors to be considered by coaches, sport scientists and performance analysts. Indeed, match-related contextual factors plays an important role in team strategies and individual pacing management due to their influence on match running performance.

## Figures and Tables

**Table 1:** Mean match running performance according to contextual factors.

	Match Location (*n* = 128)		Quality of Opposition (*n* = 128)			Match Outcome (*n* = 128)		
Measures	Away (*n* = 60)	Home (*n* = 68)	Low-Rank (*n* = 36)	Medium-Rank (*n* = 41)	High-Rank (*n* = 51)	Lose (*n* = 61)	Draw (*n* = 36)	Win (*n* = 31)
TD (km)	10.91 ± 0.83	10.95 ± 0.81	10.90 ± 0.79	10.86 ± 0.73	10.99 ± 0.91	10.89 ± 0.84	10.92 ± 0.78	11.00 ± 0.85
AvS (m · min^−1^)	0.63 ± 0.23	0.66 ± 0.25	0.59 ± 0.24	0.62 ± 0.24	0.69 ± 0.24	0.064 ± 0.23	0.66 ± 0.28	0.64 ± 0.24
rHSR (m)	68.62 ± 15.23	64.17 ± 20.41	69.94 ± 15.73	61.00 ± 19.95	68.57 ± 16.91	67.90 ± 15.41	61.56 ± 21.27	69.61 ±17.64
SPR (n)	88.74 ± 23.48	81.32 ± 23.60	85.53 ± 20.39	84.41 ± 26.61	85.75 ± 23.93	87.29 ± 20.41	78.08 ± 23.89	89.58 ± 28.21
ACC (n)	40.32 ± 13.48	42.03 ± 15.33	39.28 ± 14.15	39.95 ± 14.09	43.37 ± 14.66	40.48 ± 13.44	42.28 ± 16.19	41.07 ± 14.19
DEC (n)	0.09 ± 0.01	0.09 ± 0.01	0.09 ± 0.01	0.09 ± 0.01	0.09 ± 0.01	0.09 ± 0.01	0.09 ± 0.01	0.09 ± 0.018

ACC—accelerations; ALL—overall independent position group; AvS—average speed; CD—central defenders; CM—central midfielders; DEC—decelerations; FB—fullbacks; FW—forwards; rHSR—relative high-speed running; SPR—sprints; TD—total distance; WM—wide midfielders.

**Table 2:** Cohen’s *d*, 95% confidence intervals and smallest worthwhile changes for the influence of match location on match running performance according to playing positions.

Variables		Playing Positions									
Measures	Inference	CD vs. FB	CD vs. WM	CD vs. CM	CD vs. FW	FB vs. WM	FB vs. CM	FB vs. FW	CM vs. WM	CM vs. FW	WM vs. FW
TD (km)	*d*	0.66	0.81	1.57	0.21	0.73	0.97	0.54	0.33	1.64	1.58
	95% CI	0.55–0.77	0.57–1.05	1.32–1.82	0.17–0.25	0.60–0.86	0.80–1.14	0.38–0.70	0.27–0.39	1.41–1.87	1.29–1.87
	SWC	0.13	0.28	0.29	0.04	0.15	0.20	0.19	0.07	0.27	0.34
AvS (m · min^−1^)	*d*	0.55	0.58	0.92	0.06	0.49	0.98	1.17	0.52	1.56	1.26
	95% CI	0.37–0.73	0.52–0.64	0.87–0.97	0.03–0.09	0.44–0.54	0.92–1.04	1.14–1.20	0.51–0.53	1.55–1.57	1.23–1.29
	SWC	0.21	0.06	0.06	0.03	0.06	0.06	0.04	0.01	0.01	0.03
rHSR (m)	*d*	0.66	0.81	1.57	0.21	0.73	0.97	0.54	0.33	1.64	1.58
	95% CI	0.50–0.82	0.78–0.84	1.52–1.62	0.12–0.30	0.70–0.76	0.96–0.99	0.42–0.57	0.19–0.47	1.62–1.66	1.55–1.61
	SWC	0.18	0.03	0.06	0.10	0.04	0.01	1.10	0.16	0.02	0.03
SPR (n)	*d*	1.38	1.29	0.51	0.43	0.79	0.59	0.79	1.21	0.15	1.39
	95% CI	1.33–1.43	1.27–1.31	0.49–0.53	0.38–0.48	0.78–0.80	0.58–0.59	0.23–1.35	1.14–1.28	0.07–0.23	1.00–1.78
	SWC	0.06	0.03	0.03	0.05	0.02	0.01	0.64	0.08	0.09	0.45
ACC (n)	*d*	0.38	0.33	0.20	0.59	0.87	0.30	0.52	0.92	0.59	0.67
	95% CI	0.51–0.61	1.27–1.37	0.56–0.70	0.48–0.49	1.92–2.16	0.55–0.78	0.67–0.85	0.97–1.01	1.60–1.71	1.04–1.08
	SWC	0.06	0.06	0.08	0.01	0.14	0.13	0.10	0.02	0.07	0.03
DEC (n)	*d*	0.56	0.56	0.63	0.49	2.04	0.66	0.76	0.99	1.65	1.06
	95% CI	0.01–1.14	1.98–2.31	1.14–1.27	0.55–0.62	1.26–1.62	0.53–0.63	0.51–0.61	1.25–1.34	1.89–1.99	0.67–1.40
	SWC	0.08	0.19	0.08	0.04	0.21	0.06	0.05	0.05	0.06	0.42

Abbreviations: ACC—accelerations; AvS—average speed; CD—central defenders; CI—confidence intervals; CM—central midfielders; d—Cohen differences; DEC—decelerations; FB—fullbacks; FW—forwards; rHSR—relative high speed running; SPR—sprints; SWC—smallest worthwhile changes; TD—total distance; WM—wide midfielders.

**Table 3:** Cohen’s *d,* 95% confidence intervals and smallest worthwhile change for the influence of quality of opposition on match running performance according to playing positions.

Variables		Playing Positions									
Measures	Inference	CD vs. FB	CD vs. WM	CD vs. CM	CD vs. FW	FB vs. WM	FB vs. CM	FB vs. FW	CM vs. WM	CM vs. FW	WM vs. FW
TD (km)	*d*	0.66	1.63	1.07	0.76	0.83	1.09	1.22	0.35	1.33	2.14
	95% CI	0.62–0.70	1.58–1.68	1.00–1.14	0.69–0.83	0.76–0.90	1.03–1.15	1.18–1.26	0.34–0.35	1.20–1.46	1.88–2.40
	SWC	0.05	0.06	0.08	0.08	0.08	0.06	0.04	0.01	0.15	0.30
AvS (m · min^−1^)	*d*	0.45	1.13	1.53	0.38	1.53	1.07	0.91	0.53	0.13	1.59
	95% CI	0.41–0.49	1.04–1.22	1.50–1.56	0.26–0.50	1.42–1.64	1.00–1.14	0.87–0.95	0.48–0.58	0.08–0.18	1.38–1.80
	SWC	0.05	0.10	0.04	0.14	0.12	0.08	0.05	0.06	0.06	0.24
rHSR (m)	*d*	0.57	0.95	1.06	0.39	0.74	0.98	0.86	0.34	1.15	1.79
	95% CI	0.46–0.68	0.83–1.07	1.01–1.11	0.33–0.45	0.71–0.77	0.96–1.00	0.73–0.99	0.33–0.35	1.14–1.15	1.77–1.81
	SWC	0.13	0.14	0.06	0.07	0.03	0.03	0.15	0.01	0.01	0.02
SPR (n)	*d*	1.39	1.93	0.51	0.72	0.93	0.62	1.09	1.28	1.74	1.79
	95% CI	1.35–1.43	1.83–2.03	0.46–0.56	0.54–0.90	0.88–0.98	0.61–0.63	1.00–1.18	1.27–1.28	1.70–1.78	0.45–3.13
	SWC	0.05	0.12	0.06	0.21	0.05	0.01	0.10	0.01	0.04	1.55
ACC (n)	*d*	0.82	1.21	0.55	0.29	1.29	0.52	0.56	0.98	1.67	1.91
	95% CI	0.71–0.93	1.04–1.38	0.52–0.58	0.28–0.30	1.16–1.42	0.49–0.55	0.50–0.62	0.95–1.01	1.64–1.70	1.76–2.06
	SWC	0.13	0.20	0.03	0.01	0.15	0.03	0.06	0.04	0.03	0.18
DEC (n)	*d*	0.92	1.78	1.44	0.62	1.42	0.85	0.42	0.42	1.27	1.06
	95% CI	0.76–1.08	1.47–2.09	1.19–1.69	0.51–0.73	1.17–1.67	0.70–1.00	0.35–0.49	0.35–0.49	1.05–1.49	0.88–1.24
	SWC	0.18	0.36	0.29	0.12	0.28	0.17	0.08	0.08	0.25	0.21

Abbreviations: ACC—accelerations; AvS—average speed; CD—central defenders; CI—confidence intervals; CM—central midfielders; d—Cohen differences; DEC—decelerations; FB—fullbacks; FW—forwards; rHSR—relative high speed running; SPR—sprints; SWC—smallest worthwhile changes; TD—total distance; WM—wide midfielders.

**Table 4:** Cohen’s *d*, 95% confidence intervals and smallest worthwhile changes for the influence of match outcome on match running performance according to playing positions.

Variables		Playing Positions									
Measures	Inference	CD vs. FB	CD vs. WM	CD vs. CM	CD vs. FW	FB vs. WM	FB vs. CM	FB vs. FW	CM vs. WM	CM vs. FW	WM vs. FW
TD (km)	*d*	1.07	0.39	0.74	0.98	1.86	0.34	1.15	1.79	1.07	0.39
	95% CI	0.81–1.33	0.09–0.69	0.45–1.03	0.97–0.99	1.79–1.93	0.26–0.42	0.98–1.32	1.78–1.79	0.90–1.24	0.22–0.56
	SWC	0.30	0.35	0.33	0.01	0.08	0.09	0.19	0.01	0.19	0.20
AvS (m · min^−1^)	*d*	0.66	1.18	1.54	0.17	0.55	0.96	0.51	0.52	0.01	1.17
	95% CI	0.47–0.85	1.12–1.24	1.49–1.59	0.10–0.24	0.49–0.61	0.90–1.02	0.41–0.61	0.51–0.53	–0.02–0.03	1.11–1.23
	SWC	0.22	0.07	0.06	0.09	0.07	0.07	0.12	0.01	0.04	0.07
rHSR (m)	*d*	2.12	2.49	0.61	1.72	0.85	0.79	1.83	1.36	1.11	0.69
	95% CI	1.97–2.27	2.38–2.60	0.58–0.64	1.69–1.75	0.82–0.88	0.77–0.81	1.81–1.85	1.21–1.51	1.07–1.15	0.63–0.75
	SWC	0.17	0.13	0.03	0.04	0.04	0.02	0.03	0.17	0.05	0.07
SPR (n)	*d*	1.69	1.51	0.96	1.31	1.25	0.48	1.02	1.23	1.95	1.68
	95% CI	1.49–1.89	1.48–1.54	0.88–1.04	1.28–1.34	1.20–1.30	0.44–0.52	0.98–1.06	1.14–1.32	1.78–2.12	1.62–1.74
	SWC	0.23	0.04	0.10	0.03	0.06	0.05	0.05	0.10	0.20	0.07
ACC (n)	*d*	0.56	1.32	0.63	0.49	2.04	0.66	0.76	0.99	1.66	1.06
	95% CI	0.53–0.59	1.19–1.45	0.60–0.66	0.46–0.52	2.00–2.08	0.63–0.69	0.74–0.78	0.91–1.07	1.64–1.68	1.00–1.12
	SWC	0.03	0.15	0.03	0.03	0.05	0.04	0.03	0.09	0.02	0.07
DEC (n)	*d*	1.07	2.14	1.21	0.59	1.44	0.58	0.56	1.29	1.94	1.03
	95% CI	1.00–1.14	1.97–2.31	1.14–1.28	0.55–0.63	1.43–1.45	0.53–0.63	0.51–0.61	1.25–1.33	1.89–1.99	1.01–1.05
	SWC	0.08	0.19	0.08	0.04	0.01	0.06	0.05	0.05	0.06	0.02

Abbreviations: ACC—accelerations; AvS—average speed; CD—central defenders; CI—confidence intervals; CM—central midfielders; d—Cohen differences; DEC—decelerations; FB—fullbacks; FW—forwards; rHSR—relative high speed running; SPR—sprints; SWC—smallest worthwhile changes; TD—total distance; WM—wide midfielders.

**Table 5:** Mean match running performance according to playing position.

Measures	CD (*n* = 36)	FB (*n* = 31)	WM (*n* = 19)	CM (*n* = 33)	FW (*n* = 9)	ALL (*n* = 128)	Follow-Up (Post Hoc’s)
TD (km)	10.42 ± 0.69	10.82 ± 0.64	11.29 ± 0.55	11.54 ± 0.76	10.27 ± 0.69	10.93 ± 0.82	CM = WM > FB > FW > CD
AvS (m · min^−1^)	0.09 ± 0.07	0.09 ± 0.01	0.09 ± 0.01	0.09 ± 0.01	0.09 ± 0.01	0.09 ± 0.01	CM = WM > FB > FW > CD
rHSR (m)	0.49 ± 0.66	0.77 ± 0.17	0.89 ± 0.21	0.58 ± 0.24	0.52 ± 0.21	0.64 ± 0.24	WM = FB > CM > FW > CD
SPR (n)	32.19 ± 10.62	46.26 ± 10.58	55.58 ± 12.20	38.73 ± 14.66	37.44 ± 12.76	41.13 ± 1.27	WM = FB > CM > FW > CD
ACC (n)	62.53 ± 17.47	66.52 ± 8.76	80.89 ± 21.59	61.67 ± 20.75	70.11 ± 5.11	66.53 ± 1.58	FB = FW = WM > CM > CD
DEC (n)	69.17 ± 14.14	83.42 ± 12.12	103.42 ± 21.57	96.42 ± 29.08	77.89 ± 11.47	41.13 ± 23.74	WM = CM > FB > FW > CD

ACC—accelerations; ALL—overall independent position group; AvS—average speed; CD—central defenders; CM—central midfielders; DEC—decelerations; FB—fullbacks; FW—forwards; rHSR—relative high-speed running; SPR—sprints; TD—total distance; WM—wide midfielders.

## Data Availability

Data is available upon request to the contact author.
